# A novel clinical model for predicting malignancy of solitary pulmonary nodules: a multicenter study in chinese population

**DOI:** 10.1186/s12935-021-01810-5

**Published:** 2021-02-17

**Authors:** Xia He, Ning Xue, Xiaohua Liu, Xuemiao Tang, Songguo Peng, Yuanye Qu, Lina Jiang, Qingxia Xu, Wanli Liu, Shulin Chen

**Affiliations:** 1 State Key Laboratory of Oncology in South China, Collaborative Innovation Center for Cancer Medicine, Guangdong Key Laboratory of Nasopharyngeal Carcinoma Diagnosis and Therapy, Sun Yat-sen University Cancer Center, 651 Dongfeng Road East, 510060 Guangzhou, People’s Republic of China; 2 Research Center for Translational Medicine, the First Affiliated Hospital, Sun Yat-sen University, 58 Zhongshan Road 2, Guangdong 510080 Guangzhou, People’s Republic of China; 3 Department of Clinical Laboratory, Affiliated Cancer Hospital of Zhengzhou University, Zhengzhou Key Laboratory of Digestive Tumor Markers, Henan 450008 Zhengzhou, People’s Republic of China; 4 Department of Radiology , Affiliated Tumor Hospital of Zhengzhou University , Henan 450008 Zhengzhou, People’s Republic of China

**Keywords:** Diagnosis, Lasso logistic regression, Malignant tumor, Prediction model, Solitary pulmonary nodules

## Abstract

**Background:**

This study aimed to establish and validate a novel clinical model to differentiate between benign and malignant solitary pulmonary nodules (SPNs).

**Methods:**

Records from 295 patients with SPNs in Sun Yat-sen University Cancer Center were retrospectively reviewed. The novel prediction model was established using LASSO logistic regression analysis by integrating clinical features, radiologic characteristics and laboratory test data, the calibration of model was analyzed using the Hosmer-Lemeshow test (HL test). Subsequently, the model was compared with PKUPH, Shanghai and Mayo models using receiver-operating characteristics curve (ROC), decision curve analysis (DCA), net reclassification improvement index (NRI), and integrated discrimination improvement index (IDI) with the same data. Other 101 SPNs patients in Henan Tumor Hospital were used for external validation cohort.

**Results:**

A total of 11 variables were screened out and then aggregated to generate new prediction model. The model showed good calibration with the HL test (P = 0.964). The AUC for our model was 0.768, which was higher than other three reported models. DCA also showed our model was superior to the other three reported models. In our model, sensitivity = 78.84%, specificity = 61.32%. Compared with the PKUPH, Shanghai and Mayo models, the NRI of our model increased by 0.177, 0.127, and 0.396 respectively, and the IDI changed − 0.019, -0.076, and 0.112, respectively. Furthermore, the model was significant positive correlation with PKUPH, Shanghai and Mayo models.

**Conclusions:**

The novel model in our study had a high clinical value in diagnose of MSPNs.

## Background

Solitary pulmonary nodules (SPNs) is a term used to describe single, round, well-circumscribed radiological opacity less than 3 cm in diameter [1]. With the widespread use of low-dose computed tomography (LDCT) screening for lung cancer, a frequently reported incidence of SPNs has shown a significantly increasing trend in recent years [2]. The detection rate of SPNs has increased from 8 to 51% [3]. In the SPNs cases, malignant SPNs (MSPNs) account for less than 10% of these nodules [4]. And the National Lung Screening Trial (NLST) found that although the rate of SPNs positivity was 25%, but 96% of the nodules evaluated in that study were benign SPNs (BSPNs) [5]. The LDCT screening, in turn, gives rise to a high number of false positive results. So, correctly identification and diagnosing MSPNs is becoming more and more important. Early diagnosis and treatment of MSPNs greatly improves the overall survival rate and prognosis of patients with lung cancer [6].

Traditionally, preoperative assessment of SPNs was based on clinicians’ and radiologists’ personal experience. Therefore, the clinical experience and judgment may not be reproducible or reliable. To overcome this issue, researchers have developed some clinical mathematical prediction models based on clinical features, or radiologic characteristics, or serum markers to diagnose MSPNs. The widely used prediction model for screening SPNs include the Mayo Clinic model [7], the Department of Veterans Affairs (VA) model [8], Peking University People’s Hospital (PKUPH) model [9], Shanghai model [10], and the Bayesian Inference Malignancy Calculator (BIMC) model [11]. Although the four models are different from one another in the features that are considered as predictive factors. However, they are all developed based on clinical and imaging features.

Recently, some laboratory test data are widely used in cancer management to aid lung cancer diagnosis. Pulmonary function test (PFT) is often considered the basis for diagnosis in many categories of pulmonary disease [12]. The impaired lung function is associated with increased risk of lung cancer [13]. Serum biomarkers are easily accessible, which are widely used to aid the traditional imaging techniques to enhance the early diagnosis of lung cancer. Serum tumor markers such as cytokeratin 19 fragment (Cyfra21-1) and carcinoembryonic antigen (CEA) are commonly used to screen for lung cancer, disease monitoring and prognosis, which are recommended by both the National Academy of Clinical Biochemistry (NACB) and European Group on Tumor Markers (EGTM) [14]. In the last years, serum microRNAs (miRNAs) had been demonstrated to have an important role in tumor microenvironment and immune regulation, miRNAs could be used as a diagnostic and prognostic tool for lung cancer [15].

Until now, combine clinical features with radiologic characteristics and laboratory test data to differentiate between BSPNs and MSPNs was not reported. Multiple laboratory tests detecting, and combined analysis of clinical features and traditional imaging are a novel approach for noninvasive detection of lung cancer. Hence, the aim of this study is to construct a novel clinical model, incorporating clinical features, radiographic characteristics and laboratory test data, to identify and diagnose MSPNs in patients with SPNs. And assess its incremental value to the PKUPH model and Mayo model for individual MSPNs estimation.

## Materials and methods

### Patient selection and data collection

We performed a retrospective analysis of SPNs patients were recruited from Sun Yat-sen University Cancer Center (Guangzhou, China) between Jan 2011 to Dec 2016 as training cohort. The training cohort was used to constructed a novel model for predicting malignancy of SPNs. Addition patients with SPNs recruited in Henan Tumor Hospital (Zhengzhou, China) from Jan 2013 to Jun 2018 were used as an external validation cohort. All patients provided written informed consent to research use. This study was approved by the Hospital Ethics Committee in Sun Yat-sen University Cancer Center and Henan Tumor Hospital. This study was conducted according to the Declaration of Helsinki. The inclusion criteria were the following: a: all patients were selected based on presence of SPNs on chest CT scan. Final diagnoses were confirmed with histopathologic diagnosis based on tissue obtained from CT-guided transthoracic needle biopsy, bronchoscopy, thoracoscopy, or surgical resection; b: ≤ 3 cm diameter solitary pulmonary nodules lesion in the lung; c: no extrapulmonary malignancy; d: complete clinical, CT image, and laboratory data, and all the data were collected from electronic medical records within 7 days at diagnosis prior to any anti-tumor activity. The authenticity of this article has been validated by uploading the key raw data onto the Research Data Deposit public platform (www.researchdata.org.cn), with the approval RDD number as RDDA2020001625.

## Clinical features, radiologic characteristics and laboratory test data

Clinical features were collected from the selected patients, including age of the patient, gender, height, weight, body mass index (BMI), smoking history, family history of cancer, symptoms (fever, cough, expectoration, sputum with blood, hemoptysis, and chest pain). radiologic characteristics including tumor site (left lobe or right lobe, upper, middle or lower), radiographic characteristics including SPNs diameter, SPNs area (length of SPNs length x width of SPNs), calcification, cavity, spiculation, pleural thickening, pleural adhesion, and pleural stretch. Laboratory test data including lung function indices (vital capacity (VC), forced expiratory volume in one second (FEV1), FEV1%, FEV1/FVC, RV/TLC, diffusion capacity for carbon monoxide (DLCO), and DLCO%,) and blood-based biomarkers (white blood cell (WBC), neutrophil (N), lymphocyte (L), monocyte (M), platelets (PLT), neutrophil/lymphocyte ratio (NLR), derived NLR (dNLR) [16]: dNLR = N/(WBC - N), lymphocyte/monocyte ratio (LMR), platelet/lymphocyte ratio (PLR), systemic immune-inflammation index (SII) [17]: SII = (PLT × N)/L, red blood cell (RBC), Hemoglobin (Hbg), alanine aminotransferase (ALT), aspartate aminotransferase (AST), ALT/AST ratio (LSR), total protein (TP), albumin (ALB), globulin (GLOB), ALB/ GLOB ratio (AGR), total bile acid (TBA), total bilirubin (TBIL), direct bilirubin (DBIL), γ-glutamyl transpeptidase (GGT), alkaline phosphatase (ALP), C-reactive protein (CRP), prognostic nutritional index (PNI) [18]: PNI = ALB (g/L) + 5 × lymphocyte count × 10^9^/L, creatinine (CRE), cystatinC (Cys-C), fibrinogen (FBG), cytokeratin 19 fragment (Cyfra21-1), carcinoembryonic antigen (CEA), and neuron-specific enolase (NSE)).

### Statistical analysis

All statistical analyses were performed using SPSS software, version 19.0 (SPSS Inc., Chicago, IL, USA) and R software version 3.6.1 (http://www.R-project.org). A least absolute shrinkage and selection operator (LASSO) [19] regression was used in the training cohort for the potential predictors to select the probability of malignant SPNs (MSPNs). The novel prediction model for predicting MSPNs was established based on the results of the LASSO regression analysis. The prediction model was evaluated on discrimination and calibration. Discrimination was assessed using receiver operating characteristic (ROC) curves were used to assess the overall discrimination ability of our model and to choose its best diagnosis cut-off value [20]. Calibration reflects the agreement between predicted probabilities from the model and observed outcomes. We used the Hosmer-Lemeshow goodness-of-fit test (HL test) to statistically determine the extent of agreement between the predicted and observed probabilities [21]. To evaluate whether the new model was informative beyond PKUPH model, Shanghai model, and Mayo model. We performed area under the ROC curve (AUC), decision curve analysis (DCA) [22], net reclassification improvement index (NRI) [23], and integrated discrimination improvement index (IDI) [23] to quantify the predictive power and the added predictive ability of our model. Nomogram (by the package of rms in R) was developed to enhance the use of our model in predicting malignancy of SPNs by combining our model, PKUPH model, Shanghai model, and Mayo model. Its performance was assessed by calibration curve in internal validation with bootstrapping (1000 bootstrap resamples) [24]. Pearson’s correlation coefficient was used to identify the relationship between our model, PKUPH model, Shanghai model, and Mayo model [25]. The difference was considered statistically significant when a P-value was less than 0.05.

### Data availability

The data are not available for public access because of patient privacy concerns but are available from the corresponding author on reasonable request approved by the institutional review boards of Sun Yat-sen University Cancer Center and Affiliated Tumor Hospital of Zhengzhou University.

## Results

### Characteristics of the training and validation cohorts

In total, 396 SPNs patients were included in this retrospective study, including 295 patients from Sun Yat-sen University Cancer Center. Clinical, CT image, and laboratory data were presented in Additional file 1: Table S1. And other 101 patients from Henan Tumor Hospital were used for external validation (Additional file 2: Table S2). The mean age (SD) of patients in the training cohort was 57.0 (11.0) years; 192 patients (65.1%) were men and 189 (64.1%) patients were diagnosed as MSPNs, including 163 (86.2%) adenocarcinoma, 17 (9.0%) squamous cell carcinoma and 9 (4.8%) others. In the external validation cohort, the amounts for adenocarcinoma, squamous cell carcinoma, and others were 60 (91.0%), 3 (4.5%), and 3 (4.5%), respectively.

### Predictors selection

**Fig. 1:**
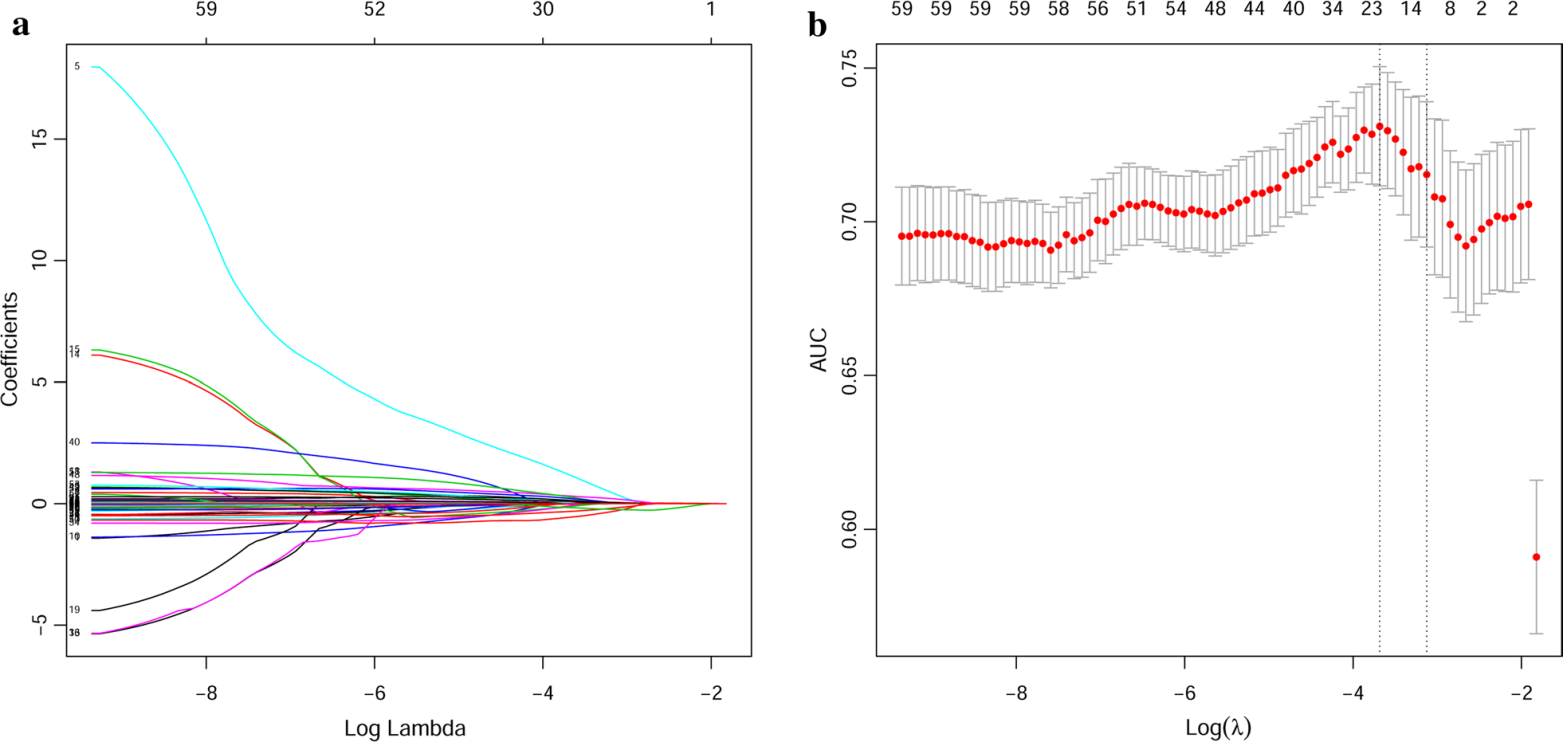
Potential predictors selection using LASSO logistic regression

To select the potential predictors for predicting malignancy of SPNs, we used LASSO logistic regression analysis. Figure 1a showed the change in trajectory of each variable was analyzed. Moreover, 10-fold cross-validation was employed for model construction, and the confidence interval under each λ was presented in Fig. 1b. According to the 1-SE criteria, we selected λ = 0.044 as the optimal value for the model, which included 11 potential predictors (age, previous cancer history, diameter, spiculation, calcification, pleural stretch, VC, FEV1, DLCO1, CEA, and NSE) with non-zero coefficients from the 63 candidate variables identified in the training cohort. The clinical and laboratory data of these selected predictors in training cohort, validation cohort, and external validation cohort were presented in Table 1.

**Table 1:** Demographics and clinical characteristics of patients in the training and validation cohort

Characteristic	Training cohort			External validation cohort	
	n = (295)			n = (101)	
	Benignancy	Malignancy		Benignancy	Malignancy
	n = 106	n = 189		n = 35	n = 66
	No.(%) or	No.(%) or		No.(%) or	No.(%) or
	Mean ± sd	Mean ± sd		Mean ± sd	Mean ± sd
Age (years)	52.0 ± 11.7	59.7 ± 9.6		51.5 ± 9.7	59.2 ± 9.3
Previous cancer history					
Yes	0 (0.0%)	9 (4.8%)		5 (14.3%)	6 (9.1%)
No	106 (100.0%)	180 (95.2%)		30 (85.7%)	60 (90.9%)
Diameter^a^ (cm)	1.8 ± 0.6	2.1 ± 0.6		1.8 ± 0.7	1.9 ± 0.6
Spiculation					
Yes	50 (52.8%)	115 (60.8%)		8 (22.9%)	21 (31.8%)
No	56 (47.2%)	74 (39.2%)		27 (77.1%)	45 (68.2%)
Calcification					
Yes	8 (7.5%)	3 (1.6%)		1 (2.9%)	3 (4.5%)
No	98 (92.5%)	186 (98.4%)		34 (97.1%)	63 (95.5%)
Pleural stretch					
Yes	24 (22.6%)	61 (32.3%)		6 (17.1%)	24 (36.4%)
No	82 (77.4%)	128 (67.7%)		29 (82.9%)	119 (63.6%)
VC (L)	3.6 ± 0.8	3.2 ± 0.7		3.3 ± 0.8	2.8 ± 0.6
FEV1 (L)	2.9 ± 0.7	2.4 ± 0.6		2.8 ± 0.7	2.3 ± 0.5
DLCO (mmol/min/kpa)	5.6 ± 3.0	6.1 ± 2.8		7.0 ± 1.4	6.0 ± 1.1
CEA (ng/mL)	2.5 ± 1.9	5.2 ± 8.3		2.1 ± 1.6	5.5 ± 17.3
NSE (ng/mL)	12.5 ± 3.8	13.4 ± 3.9		13.1 ± 4.0	13.8 ± 4.0

a: The maximum diameter of SPNs;

Abbreviations: sd: standard deviation; VC: vital capacity; FEV1: forced expiratory volume in one second; DLCO: diffusion capacity for carbon monoxide; CEA: carcinoembryonic antigen; NSE: neuron-specific enolase

### Construction and evaluation of the novel prediction model

For predicting each individual patient’s malignancy risk, the risk score was calculated for each patient with the following formula:

Risk score = − 1.137 + (0.036*age) + (0.380*previous cancer history) + (0.195*diameter) + (0.016* spiculation) − (0.290*calcification) + (0.026*pleural stretch) − (0.168*VC) − (0.236*FEV1) + (0.052*DLCO1) + (0.018*CEA) + (0.004*NSE).

Subsequently, we used the following formulas to calculate the probability of malignancy: probability (P) = e^risk score^ /(1 + e^risk score^), where e is the natural logarithm, the values for the continuous variables were medical recorded; the value for the previous cancer history, spiculation, calcification, pleural stretch, equals 1 if the element exists, and 0 otherwise.

Finally, the calibration of model was analyzed using HL test. The new prediction model showed good calibration with the HL test (P = 0.964, Additional file 3: Figure S1A). The AUC for the novel model was 0.768 (95% CI: 0.716–0.815), a P value of 0.58 was ultimately selected as a cut-off point and P values > 0.573 should be considered a malignant disease. The sensitivity of this model for the training cohort was 78.84% (72.3–84.4%), specificity = 61.32% (51.4–70.6%), positive likelihood ratio (LR+) = 2.04, and negative likelihood ratio (LR−) = 0.35.

### Validation of the novel prediction model

The performance of the novel prediction model was validated in the external validation cohort. According to the formula constructed in the training cohort, a risk score and probability of malignancy were calculated for each patient in the validation set. Then the discrimination and the calibration of the model were assessed using ROC, calibration curve, and the HL test were performed. In the external validation cohort, the AUC was 0.718 (95 % CI: 0.620–0.803), the sensitivity, specificity, LR+, and LR− of model was 81.82, 40.00%, 1.36, and 0.45. In addition, calibration curve and HL test reflected the new model had a high accuracy of the model for predicting MSPNs in the external validation cohort (P = 0.950, Additional file 3: Figure S1B).

#### Assessment the performance of our model, PKUPH model, Shanghai model, and Mayo model for SPNs screening using ROC analysis, DCA, NRI and IDI

**Fig. 2:**
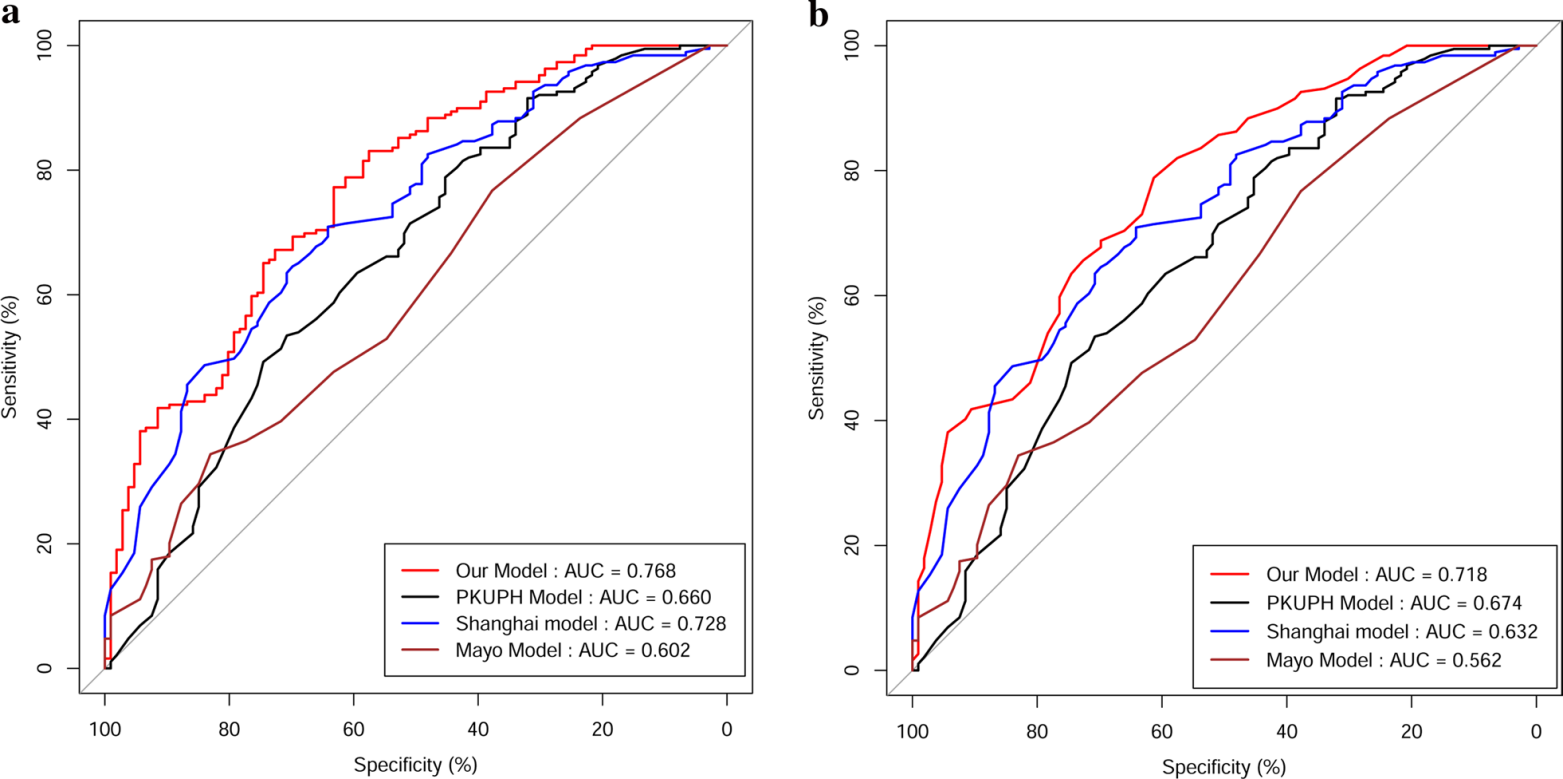
ROC comparison for the three models analyzed in training cohort (**a**), and external validation cohort (**b**), respectively

The data for training, validation and external validation cohorts were substituted into our proposed model, PKUPH model, Shanghai model, and Mayo model to generate the respective ROC curves (Fig. 2; Table 2). For the training cohort, the AUC of the three models was 0.768, 0.659, 0.728, and 0.602, respectively. The AUC of our model was higher than the PKUPH model (P < 0.001), Shanghai model (P = 0.180), and Mayo model (P < 0.001). In the external validation cohort, the AUC of the four models was 0.718, 0.674, 0.632, and 0.562, respectively. The AUC of our model was also higher than the PKUPH model (P = 0.404), Shanghai model (P = 0.048), and Mayo model (P = 0.007).

**Table 2:** Comparison of the area under the ROC curves (AUCs) of four models analyzed in this study

Models	AUCs	95% CI	P value
For training cohort			
Our model	0.768	0.716–0.815	
PKUPH model	0.659	0.602–0.713	
Shanghai model	0.728	0.674–0.778	
Mayo model	0.602	0.544–0.659	
Our model vs. PKUPH model			< 0.001
Our model vs. Shanghai model			0.180
Our model vs. Mayo model			< 0.001
For external validation cohort			
Our model	0.718	0.620–0.803	
PKUPH model	0.674	0.574–0.764	
Shanghai model	0.632	0.530–0.726	
Mayo model	0.562	0.460–0.661	
Our model vs. PKUPH model			0.404
Our model vs. Shanghai model			0.048
Our model vs. Mayo model			0.007

*ROC* receiver operating characteristic,* AUCs* areas under the curve,* CI* confidence interval

**Fig. 3:**
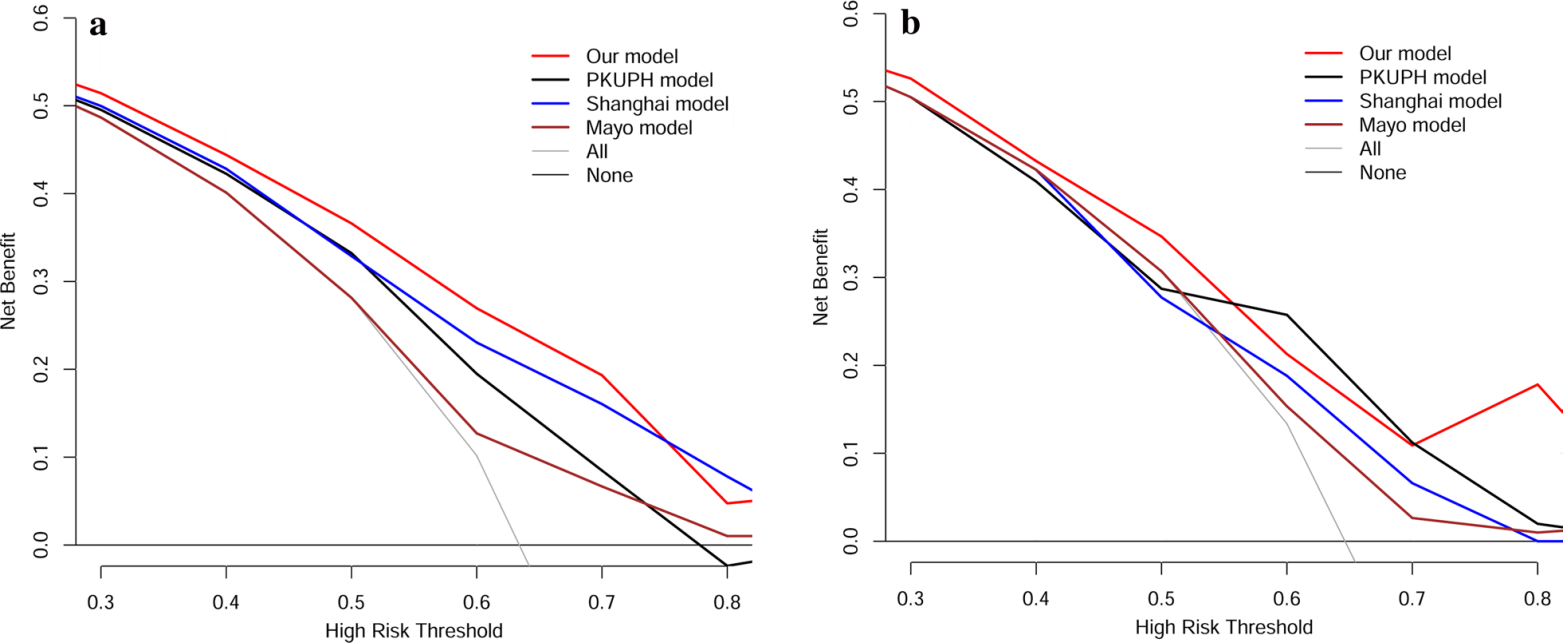
Decision curve analysis for the four models analyzed

DCA was employed to evaluate the clinical utility of the four models in the training and external validation cohorts (Fig. 3). The x-axis of the decision curve was the threshold of the predicted probability using the models to classify MSPNs patients and BSPNs patients. The y-axis shows the clinical decision net benefit for patients based on the classification result in this threshold. The decision curves of the treat-all scheme and the treat-none scheme were used as references in the decision curve analysis. Our model (red) showed had a higher overall net benefit than PKUPH model (black), Shanghai model (blue), and Mayo model (brown) both in the training and external validation cohorts. The application of our model was associated with reasonably good clinical utility across the three data.

The improvement in the predictive accuracy of our proposed model as compared to the PKUPH model and Mayo model, which was estimated by calculating the NRI and IDI in the training and external validation cohorts (Table 3). Comparing our model to PKUPH model, Shanghai model, and Mayo model, the changed in NRIs of the training and external validation cohorts were 0.177 (P = 0.005) and − 0.035 (P = 0.726), 0.127 (P = 0.058) and 0.027 (P = 0.769), 0.396 (P < 0.001) and 0.249 (P = 0.008), respectively. The changed in IDIs of the training and external validation cohorts were − 0.019 (P = 0.433) and − 0.043 (P = 0.341), − 0.076 (P = 0.005) and − 0.017 (P = 0.709), 0.112 (P < 0.001) and 0.086 (P < 0.001), respectively. These results indicated that the new model could supplement the deficiencies of the two models in predicting MSPNs.

**Table 3:** The NRI and IDI were used to assess reclassification performance and improvement in discrimination of our novel prediction model

	NRI	P Value	IDI	P Value
Training cohort				
Our model vs. PKUPH model	0.177	0.005	− 0.019	0.433
Our model vs. Shanghai model	0.127	0.058	− 0.076	0.005
Our model vs. Mayo model	0.396	< 0.001	0.112	< 0.001
External validation cohort				
Our model vs. PKUPH model	-0.035	0.726	− 0.043	0.341
Our model vs. Shanghai model	0.027	0.769	− 0.017	0.709
Our model vs. Mayo model	0.249	0.008	0.086	< 0.001

*NRI* net reclassification improvement index,* IDI* integrated discrimination improvement index

#### Comparison of the sensitivity, specificity, positive likelihood ratio, negative likelihood ratio of the four models analyzed in this study

Comparison of the sensitivity, specificity, LR+, LR− of the three models in the four independent cohorts of patients (Additional file 4: Table S3). The threshold of our model and Shanghai model was 0.58 and 0.67, respectively. And the threshold of PKUPH model and Mayo model were used literature reports as 0.463 and 0.10, respectively. In the training cohort, the performance of our model were: sensitivity: 78.84 % (95 % CI: 72.3–84.4%); specificity: 61.32% (95% CI: 51.4–70.6%); LR+: 2.04 (95% CI: 1.7–2.4); and LR−: 0.35(95% CI: 0.2–0.5); for PKUPH model, sensitivity was 85.19% (95% CI: 79.3–90.4%), specificity was 34.91% (95% CI: 25.9–44.8%); LR+: 1.31 (95% CI: 1.0–1.7); and LR−: 0.42 (95% CI: 0.3–0.6); for Shanghai model, sensitivity was 70.9% (95 % CI: 63.9–77.3%), specificity was 87.74% (95% CI: 79.9–93.3%); LR+: 2.16 (95% CI: 1.7–2.3); and LR−-: 0.45 (95% CI: 0.3–0.6); for Mayo model, sensitivity was 26.46% (95% CI: 20.3 –33.3%), specificity was 87.74% (95% CI: 79.9 –93.3%); LR+: 2.16 (95% CI: 1.7–2.8); and LR-: 0.84 (95% CI: 0.5–1.4). The specificity our model were better than PKUPH model, whereas the sensitivity was lower than PKUPH model, and the sensitivity of our model had a good performance than Shanghai model and Mayo model, but the specificity was worse than Shanghai model and Mayo model. There had inconsistent results in the external validation cohorts. Comparison of the four models at their respective thresholds in the two cohorts were inconclusive: each model has its own merits and demerits in predicting MSPNs.

#### Building and validating combined predictive nomogram

**Fig. 4:**
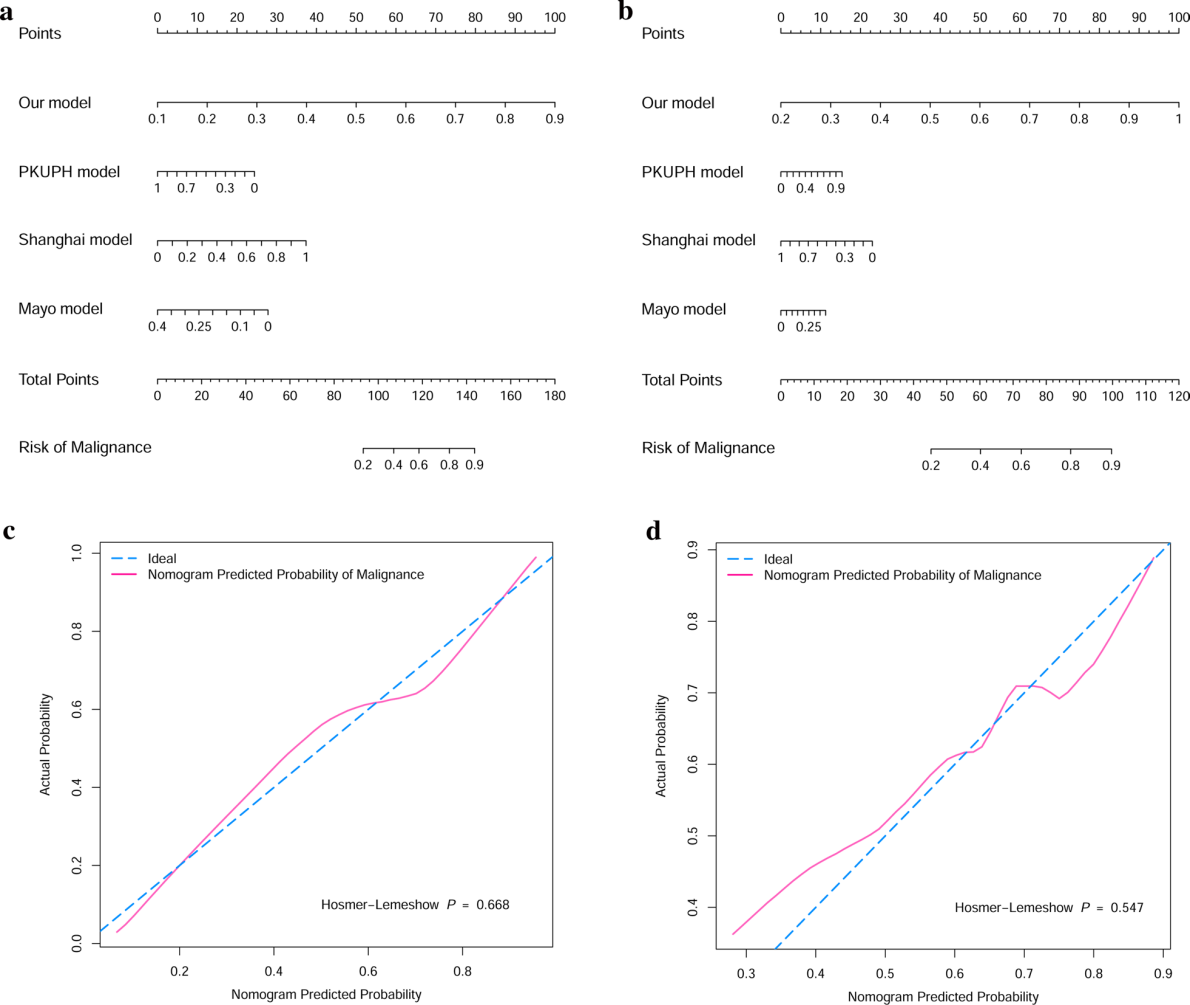
The nomograms (**a**, **b**) were used to estimate malignant SPNs, along with the calibration plot (**c**, **d**) for the nomograms in training cohort and external validation cohort, respectively

In order to combine the merits of each model in predicting MSPNs, a combined nomogram was constructed from our model, PKUPH model, Shanghai model, and Mayo model, to predict malignancy of SPNs in training cohort and external validation cohort (Fig. 4a, b respectively). Each model was assigned a point. As an example, locate our model risk score, draw a line straight upward to the “Points” axis to determine how many points associated with that model risk score. Repeat the process for each model, sum the points achieved for each covariate, and locate the sum on the “Total Points” axis. Final draw a line straight down to find the patient’s risk of malignance. The AUC of combined nomogram was 0.789 for the training set, and an AUC of 0.735 for the external validation set, which were higher than those models alone. Then the calibration curves for the probability of malignancy were used to assess the agreement between the predicted and actual observation in training cohort, validation cohort, and external validation cohort (Fig. 4c, d respectively). The calibration plots showed a good match between the prediction by nomogram and actual observation. All the results revealed the improvement of SPNs discrimination using the combined nomogram.

#### The correlation between the novel prediction, PKUPH, Shanghai, and Mayo models

**Fig. 5:**
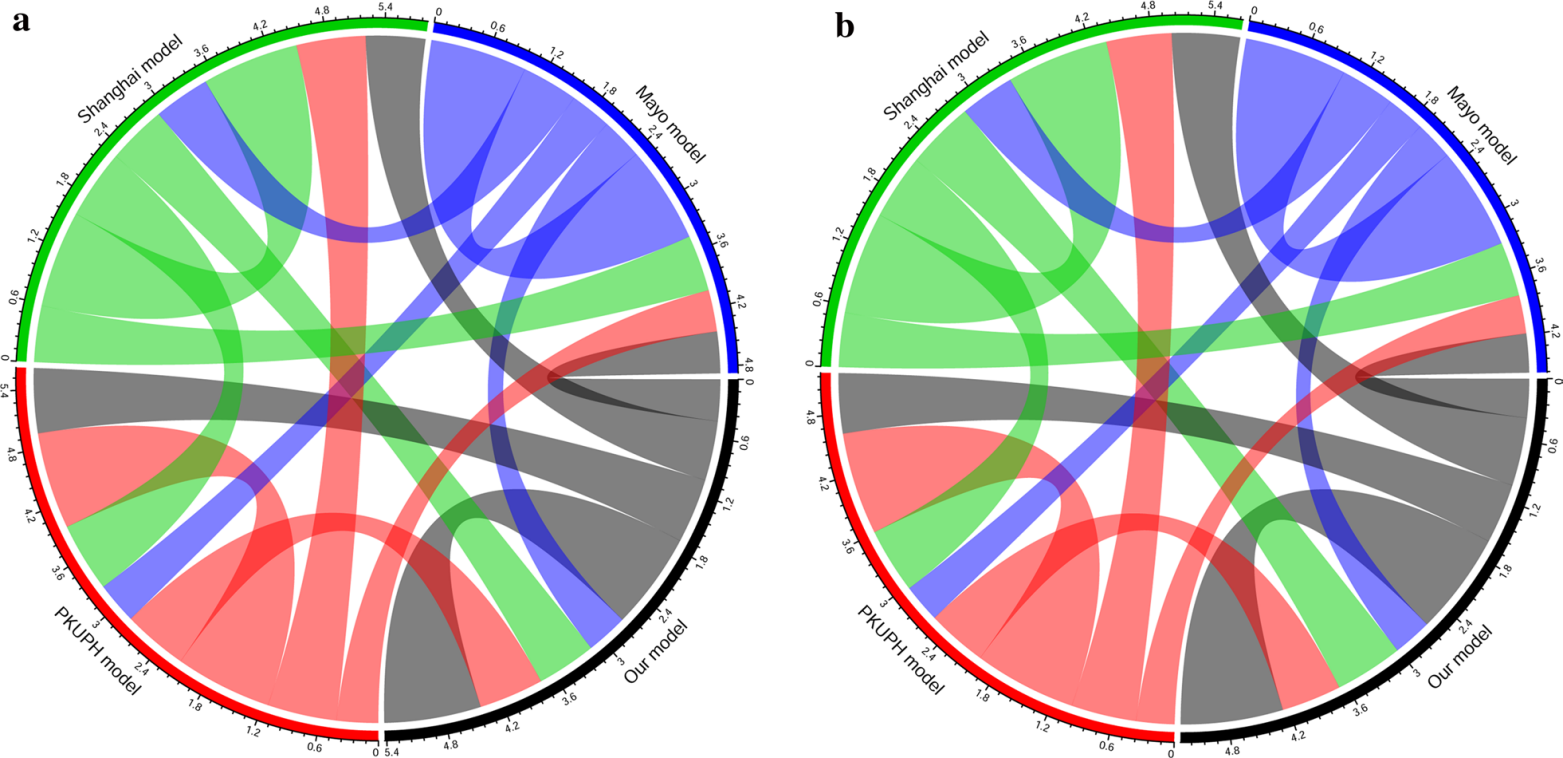
The correlations between our model, PKUPH model, Shanghai model, and Mayo model in training cohort (**a**) and external validation cohort (**b**), respectively

Figure 5 and Additional file 5: Table S4 showed the correlations between the novel prediction model, PKUPH model, and Mayo model in training cohort (A) and external validation cohort (B). Pearson’s correlation coefficients (PCC) was computed to determine the interrelationship between the three models. The results revealed that the new prediction model was significantly and positively correlated with PKUPH model (PCC: training cohort: 0.669, P < 0.001; external validation cohort: 0.586, P < 0.001), Shanghai model (PCC: training cohort: 0.613, P < 0.001; external validation cohort: 0.665, P < 0.001), and Mayo model (PCC: training cohort: 0.429, P < 0.001; external validation cohort: 0.379, P < 0.001), indicating that our analysis results had credible prediction value.

## Discussion

In this study, we conducted retrospective analysis of individual clinical features, image and laboratory data of 396 newly diagnosed SPNs patients in two cancer centers. Then a novel prediction model in predicting MSPNs was developed by using Lasso regression analysis. We compared the performance of the novel model with PKUPH model, Shanghai model, and Mayo model. The identified novel prediction model successfully classified the SPNs patients into BSPNs and MSPNs, and the new model had better ability than the three existing models in predicting MSPNs. The results were also validated with external validation cohorts, suggesting the reproducibility and reliability of the developed prediction model.

Using Lasso regression analysis, we found 11 predictors (age, previous cancer history, diameter, spiculation, calcification, pleural stretch, VC, FEV1, DLCO1, CEA, and NSE) from 63 candidate variables. Among the predictors, age [26], previous cancer history [7, 10], diameter [27], spiculation [10], calcification [28], FEV1 [13], CEA [29] and NSE [30] had been reported before. The remaining predictors that were identified in our study but not been reported in other studies, the probably because previously reported models did not incorporate these potential predictors for analysis. Thus, whether these predictors could really be used to predict MSPNs, which required more follow-up clinical studies to confirm the results.

We compared the predictive accuracy of the prediction model with PKUPH model, Shanghai model, and Mayo model. ROC curve showed that the AUC of our model was higher than PKUPH model, Shanghai model, and Mayo model all in training and external validation cohorts. The DCA also showed our model was good performance in MSPNs prediction than other three models in the two cohorts. The results of sensitivity, specificity, NRI, and IDI indicated that our model could supplement the deficiencies of the other models in predicting MSPNs. Therefore, these results may support the potential use of our model as a useful tool to help clinicians to identify and diagnose MSPNs in patients with SPNs.

Compared to the previous reported models, our study had several strengths. (1) Combined clinical features with radiologic characteristics and laboratory test data to differentiate between BSPNs and MSPNs had not been reported. This study was the first to establish a prediction model in predicting MSPNs by integrating clinical features, radiologic characteristics and laboratory test data, which could combine their individual advantages to achieve a better prediction model. (2) All models (Mayo model, VA model, and BIMC model) except the PKUPH model and Shanghai model were developed using North American or European populations. In this retrospective study including 396 SPNs patients came from two cancer centers in China. The prediction model was developed in the training cohort from 63 candidate variables and validated in the external validation cohorts. Therefore, the advantages of our study were its large SPNs sample size and inclusion of a Chinese cohort. (3) The Lasso regression analysis was utilized to select predictors and build a prediction model. The method enabled to handle the multi-collinearity problems, screen overall variables, and adjust for model’s over fitting and avoid extreme predictions. This statistical method could improve the predictive accuracy, and it had been applied in many research [31–33]. (4) In this study, the number of SPNs patients were less than Shanghai model, however, the candidate variables we analyzed were far more than it. In addition, we adopt Lasso regression analysis to select predictors, and the predictive performance was evaluated using ROC, DCA, NRI, and IDI, these statistical methods were also better than it. (5) Comparison the diagnostic accuracy and discriminative ability of the novel prediction model with PKUPH, Shanghai, and Mayo models using multiple methods including ROC analysis, DCA, NRI and IDI in the same data, making it was credible evidence supporting our analysis results. (6) In order to combine the merits of the four models in predicting MSPNs, a combined easy-to-use nomogram was constructed from the three models, and the results showed the nomogram could improve the diagnostic accuracy and agreement in MSPNs and BSPNs, and then optimize treatment in this clinical setting.

There also had several drawbacks of this study should be considered. Firstly, this was a retrospective analysis and selection bias might exist. Secondly, the sensitivity and specificity of our model was not very high, in the future, we intend to incorporate molecular markers to develop a model that could improve sensitivity and specificity in identifying MSPNs among the SPNs. Thirdly, the blood-based predictive markers in this study included only common biomarkers in clinical routine laboratory testing while other potential predictive biomarkers such as miRNAs [34], genome-wide changes in DNA methylation [35], proteomic profile in serum [36], autoantibodies or tumor-associated antigens [37] were not evaluated. Finally, although this study was a multicenter study in Chinese population, further research was still needed to fully validate the model before it can be used to clinical application.

## Conclusions

In summary, we had for the first time developed a novel prediction model by integrating clinical features, radiologic characteristics and laboratory test data, which was more accurate than three previously described models and was able to identify MSPNs from SPNs. In addition, incorporating the novel model, PKUPH model, Shanghai model, and Mayo model into a nomogram could reinforce a diagnosis of MSPNs in patients with SPNs. Nevertheless, undertaking a prospective study to further validate the model for predicting MSPNs in a large population-based LDCT screening positive setting was required.

## Supplementary Information

**Additional file 1: Table S1.** Demographics and clinical characteristics of patients from Sun Yat-sen University Cancer Center.

**Additional file 2: Table S2.** Demographics and clinical characteristics of patients from Henan Tumor Hospital.

**Additional file 3: Figure S1.** The calibration curves for the novel model in training cohort (A) and external validation cohort (B), respectively.

**Additional file 4: Table S3.** Comparison of the sensitivity, specificity, positive likelihood ratio, negative likelihood ratio of the four models analyzed in this study.

**Additional file 5: Table S4.** The correlation between our model, PKUPH model, Shanghai model, and Mayo model.

## Data Availability

The datasets analyzed during the current study are not publicly available due to patient privacy concerns, but are available from the corresponding author on reasonable request.

## Abbreviations

AUC: The areas under ROC curve
BMI: Body mass index
WBC: White blood cell
NLR: Ntrophil/lymphocyte ratio
PLR: Platelet/lymphocyte ratio
ALB: Albumin
ALT: Alanine transaminase
AST: Aspartate aminotransferase
SLR: AST/ALT ratio
ALP: Alkaline phosphatase
CRP: C-reactive protein
CAR: C-reactive protein/albumin ratio
LDH: Lactic dehydrogenase
GGT: Glutamyl transpeptidase
TBIL: Total bilirubin
DBIL: Direct bilirubin
PNI: Prognostic nutritional index
PCC: Pearson's correlation coefficient
IQR: Interquartile range
VC: Vital capacity
FEV1: Forced expiratory volume in one second
DLCO: Diffusion capacity for carbon monoxide
CEA: Carcinoembryonic antigen
NSE: Neuron-specific enolase
