# Supplementary material for: A novel clinical model for predicting malignancy of solitary pulmonary nodules: a multicenter study in chinese population
Source: Cancer Cell Int. 2021 Feb 17;21:115. doi: 10.1186/s12935-021-01810-5 (PMC7890629; doi:10.1186/s12935-021-01810-5)
Supplement: Supplementary file 1 — Additional file 1: Table S1. Demographics and clinical characteristics of patients from Sun Yat-sen University Cancer Center. [file 12935_2021_1810_MOESM1_ESM.docx]

**Supplement Table 1.** Demographics and clinical characteristics of patients from Sun Yat-sen University Cancer Center

| Variable | Training cohort (n = 295) | |
| --- | --- | --- |
|  | Benignancy (n = 106)  No. (%) or Mean ± sd | Malignancy (n = 189)  No. (%) or Mean ± sd |
| Characteristics |  |  |
| Gender |  |  |
| Male | 29 (27.4%) | 74 (39.2%) |
| Female | 77 (72.6%) | 115 (60.8%) |
| Age (years) | 52.0 ± 11.7 | 59.7 ± 9.6 |
| Height | 165.3 ± 8.0 | 163.8 ± 7.4 |
| Weight | 62.8 ± 10.5 | 61.6 ± 9.5 |
| BMI (kg/m^2^) | 22.9 ± 3.1 | 23.0 ± 3.1 |
| Smoke |  |  |
| Yes | 45 (42.5%) | 89 (47.1%) |
| No | 61 (57.5%) | 100 (52.9%) |
| Family history of cancer |  |  |
| Yes | 19 (17.9%) | 30 (15.9%) |
| No | 87 (82.1%) | 159 (84.1%) |
| Previous cancer history |  |  |
| Yes | 0 (0.0%) | 9 (4.8%) |
| No | 106 (100.0%) | 180 (95.2%) |
|  |  |  |
| Symptoms |  |  |
| Fever |  |  |
| Yes | 2 (1.9%) | 5 (2.6%) |
| No | 104 (98.1%) | 184 (97.4%) |
| Cough |  |  |
| Yes | 35 (33.0%) | 72 (38.1%) |
| No | 71 (67.0%) | 117 (61.9%) |
| Expectoration |  |  |
| Yes | 26 (24.5%) | 52 (27.5%) |
| No | 80 (75.5%) | 137 (72.5%) |
| Sputum with blood |  |  |
| Yes | 10 (9.4%) | 19 (10.1%) |
| No | 96 (90.6%) | 170 (89.9%) |
| Hemoptysis |  |  |
| Yes | 4 (3.8%) | 4 (2.1%) |
| No | 102 (96.2%) | 185 (97.9%) |
| Chest pain |  |  |
| Yes | 16 (15.1%) | 22 (11.6%) |
| No | 90 (84.9%) | 167 (88.4%) |
|  |  |  |
| Image data |  |  |
| Lung lobe |  |  |
| Left | 47 (44.3%) | 91 (48.1%) |
| Right | 59 (55.7%) | 98 (51.9%) |
| Position |  |  |
| Upper | 57 (53.8%) | 104 (55.0%) |
| Middle | 7 (6.6%) | 22 (11.7%) |
| Lower | 42 (39.6%) | 63 (33.3%) |
| Diameter^a^ (cm) | 1.8 ± 0.6 | 2.1 ± 0.6 |
| [SPNs area](https://xueshu.baidu.com/usercenter/paper/show?paperid=04066176ea30310666d3922d30e11816&site=xueshu_se) ^b^(cm^2^) | 3.0 ± 2.0 | 3.7 ± 2.1 |
| Clear border |  |  |
| Yes | 42 (39.6%) | 77 (40.7%) |
| No | 64 (60.6%) | 112 (59.3%) |
| Calcification |  |  |
| Yes | 8 (7.5%) | 3 (1.6%) |
| No | 98 (92.5%) | 186 (98.4%) |
| Cavity |  |  |
| Yes | 3 (2.8%) | 14 (7.4%) |
| No | 103 (97.2%) | 175 (92.6%) |
| Spiculation |  |  |
| Yes | 50 (47.2%) | 115 (60.8%) |
| No | 56 (52.8%) | 74 (39.2%) |
| Pleural thickening |  |  |
| Yes | 17 (16.0%) | 25 (13.2%) |
| No | 89 (84.0%) | 164 (86.8%) |
| Pleural adhesion |  |  |
| Yes | 14 (13.2%) | 37 (19.6%) |
| No | 92 (86.8%) | 152 (80.4%) |
| Pleural stretch |  |  |
| Yes | 24 (22.6%) | 61 (32.3%) |
| No | 82 (77.4%) | 128 (67.7%) |
|  |  |  |
| Laboratory data |  |  |
| VC (L) | 3.6 ± 0.8 | 3.2 ± 0.7 |
| FEV1 (L) | 2.9 ± 0.7 | 2.4 ± 0.6 |
| FEV1% | 98.6 ± 16.7 | 99.9 ± 55.0 |
| FEV1/FVC | 80.3 ± 9.3 | 78.0 ± 8.1 |
| RV/TLC | 37.4 ± 28.9 | 42.0 ± 30.2 |
| DLCO (mmol/min/kpa) | 5.6 ± 3.0 | 6.1 ± 2.8 |
| DLCO% | 96.5 ± 18.4 | 96.0 ± 20.2 |
| WBC (10^9^/L) | 6.7 ± 1.5 | 6.7 ± 1.8 |
| Neutrophil (10^9^/L) | 3.9 ± 1.3 | 4.1 ± 1.4 |
| Lymphocyte (10^9^/L) | 2.0 ± 0.7 | 2.0 ± 0.6 |
| Monocyte (10^9^/L) | 0.5 ± 0.2 | 0.5 ± 0.2 |
| PLT (10^9^/L) | 223.4 ± 65.9 | 229.1 ± 66.2 |
| NLR | 2.1 ± 0.8 | 2.3 ± 1.0 |
| dNLR | 1.5 ± 0.6 | 1.7 ± 0.7 |
| LMR | 4.7 ± 2.0 | 4.8 ± 1.8 |
| PLR | 116.8 ± 37.6 | 126.6 ± 51.0 |
| SII | 459.4 ± 217.5 | 532.4 ± 330.3 |
| RBC (10^12^/L) | 4.8 ± 0.5 | 4.7 ± 0.5 |
| Hbg (g/L) | 142.4 ± 13.4 | 140.6 ± 13.1 |
| ALT (U/L) | 25.0 ± 15.3 | 21.8 ± 10.7 |
| AST (U/L) | 21.5 ± 7.3 | 20.8 ± 5.8 |
| LSR | 1.1 ± 0.4 | 1.0 ± 0.3 |
| TP (g/L) | 72.5 ± 5.7 | 71.8 ± 4.7 |
| ALB (g/L) | 44.3 ± 3.3 | 43.3 ± 2.8 |
| GLOB (g/L) | 28.3 ± 4.0 | 28.5 ± 3.5 |
| AGR | 1.6 ± 0.2 | 1.5 ± 0.2 |
| TBA (umol/L) | 4.8 ± 6.0 | 4.3 ± 3.6 |
| TBIL (umol/L) | 12.4 ± 5.1 | 11.8 ± 4.9 |
| DBIL (umol/L) | 3.8 ± 1.7 | 3.4 ± 1.4 |
| GGT (U/L) | 34.8 ± 30.5 | 29.6 ± 24.8 |
| ALP (U/L) | 73.8 ± 22.1 | 77.7 ± 26.5 |
| CRP (mg/L) | 3.2 ± 8.7 | 2.6 ± 4.8 |
| PNI | 54.3 ± 4.3 | 53.1 ± 4.2 |
| CRE (umol/L) | 74.2 ± 17.9 | 73.0 ± 16.8 |
| Cys-C (mg/L) | 0.9 ± 0.2 | 1.0 ± 0.2 |
| FBG (g/L) | 2.9 ± 0.7 | 2.9 ± 0.7 |
| Cyfra21-1 (ng/mL) | 2.7 ± 1.2 | 3.1 ± 1.5 |
| CEA (ng/mL) | 2.5 ± 1.9 | 5.2 ± 8.4 |
| NSE (ng/mL) | 12.5 ± 3.8 | 13.4 ± 3.9 |

a: The SPNs maximum diameter;

b: SPNs area was defined as the length of SPNs length x width of SPNs.
