# Supplementary material for: A novel clinical model for predicting malignancy of solitary pulmonary nodules: a multicenter study in chinese population
Source: Cancer Cell Int. 2021 Feb 17;21:115. doi: 10.1186/s12935-021-01810-5 (PMC7890629; doi:10.1186/s12935-021-01810-5)
Supplement: Supplementary file 2 — Additional file 2: Table S2. Demographics and clinical characteristics of patients from Henan Tumor Hospital. [file 12935_2021_1810_MOESM2_ESM.docx]

**Supplement Table 2.** Demographics and clinical characteristics of patients from Henan Tumor Hospital

| Variable | External validation cohort (n = 101) | |
| --- | --- | --- |
|  | Benignancy (n = 35)  No. (%) or Mean ± sd | Malignancy (n = 66)  No. (%) or Mean ± sd |
| Characteristics |  |  |
| Gender |  |  |
| Male | 20 (57.1%) | 21 (31.8%) |
| Female | 15 (42.9%) | 45 (68.2%) |
| Age (years) | 51.5 ± 9.7 | 59.2 ± 9.3 |
| Smoke |  |  |
| Yes | 13 (37.1%) | 18 (27.3%) |
| No | 22 (62.9%) | 48 (72.7%) |
| Family history of cancer |  |  |
| Yes | 9 (25.7%) | 16 (24.2%) |
| No | 26 (74.3%) | 50 (75.8%) |
| Previous cancer history |  |  |
| Yes | 5 (14.3%) | 6 (9.1%) |
| No | 30 (85.7%) | 60 (90.9%) |
|  |  |  |
| Symptoms |  |  |
| Fever |  |  |
| Yes | 1 (2.9%) | 2 (3.0%) |
| No | 34 (97.1%) | 64 (97.0%) |
| Cough |  |  |
| Yes | 9 (25.7%) | 16 (24.2%) |
| No | 26 (74.3%) | 50 (75.8%) |
| Expectoration |  |  |
| Yes | 7 (20.0%) | 11 (16.7%) |
| No | 28 (80.0%) | 55 (83.3%) |
| Sputum with blood |  |  |
| Yes | 2 (5.7%) | 5 (7.6%) |
| No | 33 (94.3%) | 61 (92.4%) |
| Hemoptysis |  |  |
| Yes | 2 (5.7%) | 4 (6.1%) |
| No | 33 (94.3%) | 62 (93.9%) |
| Chest pain |  |  |
| Yes | 4 (11.4%) | 9 (13.6%) |
| No | 31 (88.6%) | 57 (86.4%) |
|  |  |  |
| Image data |  |  |
| Lung lobe |  |  |
| Left | 11 (31.4%) | 26 (39.4%) |
| Right | 24 (68.6%) | 40 (60.6%) |
| Position |  |  |
| Upper | 20 (57.1%) | 35 (53.0%) |
| Middle | 3 (8.6%) | 6 (9.1%) |
| Lower | 12 (34.3%) | 25 (37.9%) |
| Diameter^a^ (cm) | 1.8 ± 0.7 | 1.9 ± 0.6 |
| [SPNs area](https://xueshu.baidu.com/usercenter/paper/show?paperid=04066176ea30310666d3922d30e11816&site=xueshu_se) ^b^(cm^2^) | 3.0 ± 1.9 | 3.0 ± 1.5 |
| Clear border |  |  |
| Yes | 14 (40.0%) | 17 (25.8%) |
| No | 21 (60.0%) | 49 (74.2%) |
| Calcification |  |  |
| Yes | 1 (2.9%) | 3 (4.5%) |
| No | 34 (97.1%) | 63 (95.5%) |
| Cavity |  |  |
| Yes | 2 (5.7%) | 4 (6.1%) |
| No | 33 (94.3%) | 62 (93.9%) |
| Spiculation |  |  |
| Yes | 8 (22.9%) | 21 (31.8%) |
| No | 27 (77.1%) | 45 (68.2%) |
| Pleural thickening |  |  |
| Yes | 5 (14.3%) | 5 (7.6%) |
| No | 30 (85.7%) | 61 (92.4%) |
| Pleural adhesion |  |  |
| Yes | 11 (31.4%) | 8 (12.1%) |
| No | 24 (68.6%) | 58 (87.9%) |
| Pleural stretch |  |  |
| Yes | 6 (17.1%) | 24 (36.4%) |
| No | 29 (82.9%) | 42 (63.6%) |
|  |  |  |
| Laboratory data |  |  |
| VC (L) | 3.3 ±0.8 | 2.8 ± 0.6 |
| FEV1 (L) | 2.8 ± 0.7 | 2.3 ± 0.5 |
| FEV1% | 94.9 ± 15.5 | 95.6 ± 17.8 |
| FEV1/FVC | 83.9 ± 7.6 | 84.1 ± 6.4 |
| RV/TLC | 40.1 ± 7.6 | 43.6 ± 6.0 |
| DLCO (mmol/min/kpa) | 7.0 ± 1.4 | 6.0 ± 1.1 |
| DLCO% | 81.1 ± 14.7 | 78.3 ± 10.9 |
| WBC (10^9^/L) | 6.5 ± 2.0 | 6.2 ± 2.2 |
| Neutrophil (10^9^/L) | 3.9 ± 1.6 | 3.8 ± 1.9 |
| Lymphocyte (10^9^/L) | 2.1 ± 1.0 | 1.9 ± 0.6 |
| Monocyte (10^9^/L) | 0.3 ± 0.2 | 0.3 ± 0.1 |
| PLT (10^9^/L) | 243.5 ± 68.0 | 225.8 ± 54.3 |
| NLR | 2.1 ± 1.6 | 2.2 ± 2.3 |
| dNLR | 1.6 ± 0.8 | 1.8 ± 1.4 |
| LMR | 7.9 ± 4.4 | 7.8 ± 2.8 |
| PLR | 128.8 ± 52.9 | 126.6 ± 49.7 |
| RBC (10^12^/L) | 4.5 ± 0.5 | 4.4 ± 0.5 |
| Hbg (g/L) | 136.8 ± 14.2 | 132.5 ± 14.5 |
| ALT (U/L) | 22.5 ± 17.7 | 18.3 ± 11.9 |
| AST (U/L) | 18.6 ± 8.2 | 19.9 ± 13.2 |
| LSR | 1.2 ± 0.5 | 1.0 ± 0.3 |
| TP (g/L) | 68.8 ± 5.9 | 68.6 ± 5.3 |
| ALB (g/L) | 43.8 ± 3.8 | 43.3 ± 3.3 |
| GLOB (g/L) | 25.0 ± 3.4 | 25.2 ± 3.4 |
| AGR | 1.8 ± 0.2 | 1.7 ± 0.2 |
| TBA (umol/L) | 8.3 ± 5.9 | 6.1 ± 4.5 |
| TBIL (umol/L) | 9.7 ± 3.6 | 12.0 ± 4.6 |
| DBIL (umol/L) | 3.4 ± 1.0 | 3.8 ± 1.3 |
| GGT (U/L) | 31.6 ± 35.3 | 25.1 ± 28.9 |
| ALP (U/L) | 76.4 ± 25.6 | 68.4 ± 21.0 |
| CRE (umol/L) | 63.2 ± 12.3 | 61.4 ± 14.4 |
| FBG (g/L) | 2.5 ± 0.6 | 2.6 ± 0.6 |
| Cyfra21-1 (ng/mL) | 2.9 ± 1.5 | 2.9 ± 1.3 |
| CEA (ng/mL) | 2.1 ± 1.6 | 5.5 ± 17.3 |
| NSE (ng/mL) | 13.1 ±4.0 | 13.8 ± 4.0 |

a: The SPNs maximum diameter;

b: SPNs area was defined as the length of SPNs length x width of SPNs.
