# Supplementary figures and images for: A novel clinical model for predicting malignancy of solitary pulmonary nodules: a multicenter study in chinese population
Source: Cancer Cell Int. 2021 Feb 17;21:115. doi: 10.1186/s12935-021-01810-5 (PMC7890629; doi:10.1186/s12935-021-01810-5)

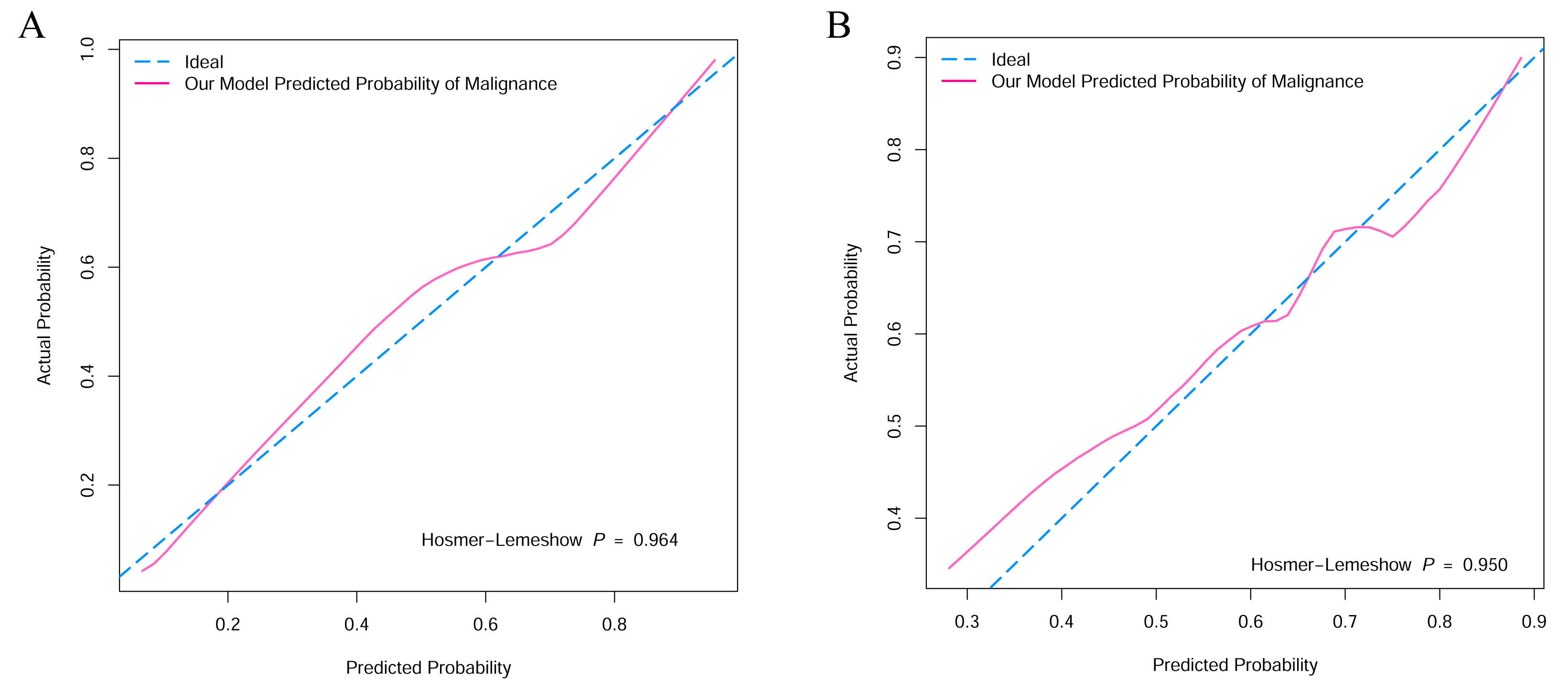

Supplement: Supplementary file 3 — Additional file 3: Figure S1. The calibration curves for the novel model in training cohort (A) and external validation cohort (B), respectively. [file 12935_2021_1810_MOESM3_ESM.tif]
