# Supplementary material for: A novel clinical model for predicting malignancy of solitary pulmonary nodules: a multicenter study in chinese population
Source: Cancer Cell Int. 2021 Feb 17;21:115. doi: 10.1186/s12935-021-01810-5 (PMC7890629; doi:10.1186/s12935-021-01810-5)
Supplement: Supplementary file 4 — Additional file 4: Table S3. Comparison of the sensitivity, specificity, positive likelihood ratio, negative likelihood ratio of the four models analyzed in this study. [file 12935_2021_1810_MOESM4_ESM.docx]

**Supplement Table 3.** Comparison of the sensitivity, specificity, positive likelihood ratio, negative likelihood ratio of the four models analyzed in this study

| Groups | Cut-off  points | Sensitivity  (95%CI) | Specificity  (95%CI) | Positive likelihood ratio (95%CI) | Negative likelihood  ratio (95%CI) |
| --- | --- | --- | --- | --- | --- |
| Training cohort |  |  |  |  |  |
| Our model | 0.58 | 78.84% (72.3%-84.4%) | 61.32% (51.4%-70.6%) | 2.04 (1.7-2.4) | 0.35 (0.2-0.5) |
| PKUPH model | 0.463 | 85.19% (79.3%-90.4%) | 34.91% (25.9%-44.8%) | 1.31 (1.0-1.7) | 0.42 (0.3-0.6) |
| Shanghai model | 0.67 | 70.9% (63.9%-77.3%) | 64.15% (54.3%-73.2%) | 1.98 (1.7-2.3) | 0.45 (0.3-0.6) |
| Mayo model | 0.10 | 26.46% (20.3%-33.3%) | 87.74% (79.9%-93.3%) | 2.16 (1.7-2.8) | 0.84 (0.5-1.4) |
|  |  |  |  |  |  |
| External validation cohort |  |  |  |  |  |
| Our model | 0.58 | 81.82 % (70.4%-90.2%) | 40.00 % (23.9%-57.9%) | 1.36 (0.9-2.1) | 0.45 (0.3-0.8) |
| PKUPH model | 0.463 | 80.30 % (68.7%-89.1%) | 42.86% (26.3%-60.6%) | 1.41 (0.9-2.1) | 0.46 (0.3-0.8) |
| Shanghai model | 0.67 | 48.48% (36.0%-61.1%) | 68.57% (50.7%-83.1%) | 1.54 (1.1-2.2) | 0.75 (0.4-1.3) |
| Mayo model | 0.10 | 10.61% (4.4%-20.6%) | 97.14% (85.1%-99.9%) | 3.71 (1.8-7.5) | 0.92 (0.1-6.4) |
