# Supplementary material for: A novel clinical model for predicting malignancy of solitary pulmonary nodules: a multicenter study in chinese population
Source: Cancer Cell Int. 2021 Feb 17;21:115. doi: 10.1186/s12935-021-01810-5 (PMC7890629; doi:10.1186/s12935-021-01810-5)
Supplement: Supplementary file 5 — Additional file 5: Table S4. The correlation between our model, PKUPH model, Shanghai model, and Mayo model. [file 12935_2021_1810_MOESM5_ESM.docx]

**Supplement Table 4.** The correlation between our model, PKUPH model, Shanghai model, and Mayo model

| Models | Correlation coefficients ^a^ | P value |
| --- | --- | --- |
| For training cohort |  |  |
| Our model vs PKUPH model | 0.669 | < 0.001 |
| Our model vs Shanghai model | 0.613 | < 0.001 |
| Our model vs Mayo model | 0.429 | < 0.001 |
|  |  |  |
| For external validation cohort |  |  |
| Our model vs PKUPH model | 0.586 | < 0.001 |
| Our model vs Shanghai model | 0.665 | < 0.001 |
| Our model vs Mayo model | 0.379 | < 0.001 |

a: Pearson's correlation coefficient.
